# Frequency and factors associated with the utilization (curative and preventive) of oral health care services among pregnant women in Kinshasa, Democratic Republic of Congo

**DOI:** 10.1038/s41405-025-00308-w

**Published:** 2025-02-14

**Authors:** Patrick Marob-Ndjock Sekele, Erick Ntambwe Kamangu, Harry-César Ntumba Kayembe, Fatiha Chandad, Jacques Ileboso Bolenge, Stephanie Mikalo Mbambi, Jean-Paul Isouradi-Bourley Sekele, Em Kazadi Kalala, Fidele Bushabu Nyimi, Pierre Zalagile Akilimali

**Affiliations:** 1 Department of Periodontology, Faculty of Dental Medicine, University of Kinshasa, Kinshasa, DR Congo; 2 Department of Basic Sciences, Faculty of Medicine, University of Kinshasa, Kinshasa, DR Congo; 3 Pool of Immunology and Microbiology, Oral Ecology Research Group. Faculty of Dentistry, Laval University, Québec, Canada; 4 Department of Pediatrics, Faculty of Medicine, Protestant University in Congo, Kinshasa, DR Congo; 5 Department of Dental and Maxillofacial Prosthesis, Faculty of Dental Medicine, University of Kinshasa, Kinshasa, DR Congo; 6 Department of Oral and Maxillofacial Surgery, Faculty of Dental Medicine, University of Kinshasa, Kinshasa, DR Congo; 7 Department of Nutrition, Kinshasa School of Public Health, Faculty of Medicine, University of Kinshasa, Kinshasa, DR Congo

**Keywords:** Dental epidemiology, Oral hygiene

## Abstract

**Background:**

The Democratic Republic of Congo (DRC) has one of the highest maternal and neonatal mortality rates in Africa. There is a growing body of evidence about the relationship between poor oral health and adverse pregnancy outcomes. However, there is a lack of information about oral health status during pregnancy in the DRC. This study aimed to identify the factors related to the utilization of oral health care services among pregnant women.

**Methods:**

A hospital-based cross-sectional study was conducted in four health facilities representing each administrative district of the city of Kinshasa, between March and May, 2021. The study population comprised pregnant women aged at least 18 or over attending antenatal care (ANC), selected using a simple random sampling technique. Data were collected using a structured questionnaire. Multivariable logistic regression was employed to identify factors associated with the outcome variable using the adjusted odds ratio (AOR) with its 95% confidence interval (95% CI) and *p* value  <  0.05.

**Results:**

A total of 5% of the 500 pregnant women who participated in the study were identified as users of oral healthcare services. 15% of pregnant women were aware of the necessity of oral health care during pregnancy, while 58% indicated that dental visits for routine and/or treatment purposes were not a priority during pregnancy. Factors associated with its utilization were knowledge of the need for oral health care during pregnancy (AOR:3.62, 95% CI: 1.42–9.26), knowledge of the importance of routine visits or dental treatment for oral diseases during pregnancy (AOR: 4.94, 95% CI: 1.70–16.73), and having experience oral health problem during the current pregnancy (AOR: 3.13, 95% CI: 1.22–8.21).

**Conclusion:**

The utilization of oral health care services during pregnancy is very low. Appropriate public health initiatives are urgently needed to facilitate collaboration between health professionals to integrate oral and dental consultations, oral health counseling, and check-ups as an essential component of routine ANC.

## Background

Pregnancy is a physiological state that is characterized by hormonal fluctuations which influence body systems and result in behavioral and/or psychological changes that require particular attention from health professionals [1–3]. It is important to note that the oral cavity is not exempt from these changes. In particular, alterations in the oral cavity have been associated with an increased risk and severity of certain pathological conditions. For instance, research has demonstrated that gingivitis, an early stage of periodontal disease, affects approximately 60–75% of pregnant women [4]. The incidence of gingivitis was also observed to be 1.81–2.2 times higher in pregnant women compared to non-pregnant women [5]. Furthermore, emerging evidence suggests a potential association between periodontal disease and an increased risk of adverse pregnancy outcomes. These include prematurity and low birth weight for the newborn, as well as preeclampsia for the mother [6]. However, further studies are required to substantiate this evidence.

To reduce the vulnerability of pregnant women to the risks of adverse pregnancy outcomes for themselves and their newborns, the utilization of oral health care services during pregnancy is crucial [1, 3, 7–9]. This can contribute to the prevention of the negative effects of periodontal disease on the maternal and fetal immune response systemically, as well as the prevention of the direct translocation of oral bacteria into the pregnant uterus, causing localized inflammation and adverse pregnancy outcomes [10]. In addition to periodontal treatment, educating pregnant women about the prevention of dental caries enables the development of attitudes and behaviors that promote women’s own oral health and that of their children in the future. Since the majority of infants and young children acquire bacteria that cause dental caries from their mothers, promoting healthy oral health behaviors may reduce the transmission of such bacteria from mothers to infants and young children, thereby delaying or preventing the onset of caries [11].

The frequency of oral health care utilization during pregnancy varies considerably between countries. A review of the literature reveals that the proportion of pregnant women who attend dental appointments ranges from 16.7 to 83% [3, 9, 10, 12]. Furthermore, in low-resource settings, the proportion of pregnant women who utilize dental services can be significantly lower [13]. Demographic and socioeconomic factors (age, marital status, parity, place of living, education level, and income), as well as psychological and behavioral factors (oral health practices, oral health and pregnancy beliefs, and health care maintenance) are likely to be associated with the utilization of oral health services during pregnancy [7, 9, 14].

It is evident that oral health care during pregnancy is a crucial component of quality antenatal care (ANC). It plays a pivotal role in reducing mortality among pregnant women and newborns, particularly in low- and middle-income countries. According to the World Health Organization (WHO), approximately 95% of all maternal deaths worldwide occur in these countries, with nearly 70% occurring in sub-Saharan Africa [15]. The estimated mortality rate in this region is 525 deaths per 100,000 live births, with 27 neonatal deaths per 1000 live births [16]. Furthermore, the maternal mortality rate is projected to reach 390 women in childbirth for every 100,000 live births by 2030, which exceeds the Sustainable Development Goals (SDGs) target [16]. The Democratic Republic of Congo (DRC) has one of the highest maternal and neonatal mortality rates in Africa, with a maternal mortality rate of over 500 deaths per 100,000 live births and a neonatal mortality rate of 27 deaths per 1000 live births, respectively [16–18].

Despite the implementation of the WHO’s cooperation strategy with the DRC (2017–2021), which aimed to improve access to operational interventions that promote health, including women’s health [19], ANC programs are often initiated late in pregnancy and implemented poorly and irregularly in the DRC [20, 21]. Furthermore, the WHO’s African Regional Strategy for Oral Health (2016–2025), which aims to ensure access to oral health care services for at least 50% of the population in need [22], presents a significant opportunity to enhance oral health check-ups, oral health promotion, and preventive supply services for pregnant women. However, there is currently a lack of accurate and up-to-date oral health information for pregnant women in the DRC. The objective of this study was to ascertain the frequency of utilization of oral health care services and examine associated factors among Congolese pregnant women.

## Methods

### Study setting and design

This cross-sectional study was conducted in Kinshasa, the capital and largest city of the DRC, from March to May, 2021. The study included four health facilities, each representing an administrative district of the city of Kinshasa: (i) the Sino-Congolese Friendship Hospital (District of Tshangu), (ii) Saint Gabriel Hospital (District of Mont-Amba), (iii) the Libikisi Reference Hospital (District of Funa), and (iv) the Maternity of Kintambo general referral hospital (District of Lukunga). The four health facilities were selected based on the following criteria: they are secondary-level institutions, have a high rate of ANC attendance, and have operational stomatology departments.

The Saint Gabriel Hospital, with 100 beds and 198 agents, provides primary health care to all sections of the population of the city of Kinshasa, starting with the poor, by providing them with a quality care package at reasonable prices, and to combat poverty. It also contributes to the practical training of the many trainees it receives [23]. The Kintambo general referral hospital, as defined by the Ministry of Public Health’s organic framework for general referral hospitals in health zones, provides a comprehensive range of healthcare services, including curative, preventive, promotional, and rehabilitative care for patients, as well as teaching and research activities [24]. The Sino-Congolese Friendship Hospital’s mission is to provide comprehensive care for patients, with a particular focus on those from Tshangu. Additionally, this 214-bed hospital aims to reduce the number of Congolese patients seeking care abroad, facilitate training for medical professionals, and engage in knowledge exchange with other medical training programs and the Chinese medical mission [25]. The Libikisi Reference Hospital is a private health facility owned by the Evangelical Mission of the Baptist Community of Congo. The facility has a total of 38 beds and an average of 150 consultations per day [26].

### Participants

This study included all pregnant women who attended ANC services at the selected health facilities during the study period. The inclusion criteria were pregnant women who: (i) were at least 18 years old, (ii) attending ANC services at the time of data collection, and (iii) provided informed consent to participate in the study. However, pregnant women who were seriously ill or had a history of mental disorders were excluded from the study.

### Sampling procedures

The sample size was calculated using the following formula:

$${{{\rm{n}}} = ({{{\rm{Z}}}}_{{{\rm{\alpha }}}/2})}^{2}\frac{{pq}\,}{{(d)}^{2}\,}$$

where *p* = estimated proportion of pregnant women using oral and dental services in Kinshasa (50% = 0.5); q = 1-*p* = 0.5 (proportion of pregnant women who do not use oral and dental services); d = degree of absolute precision desired: 0.05 (d = 5%); α = error of the first type: 0.05; Z_α/2_ = 95% confidence coefficient (1.96)²; *n* = minimum sample size. The required number for the largest sample size was 384, and 10% of the total sample size was added to compensate for the non-response rate. Finally, 500 pregnant women were included.

The sample was allocated proportionally to the average number of pregnant women who had received ANC in each health facility during the two months preceding the data collection period: Sino-Congolese Friendship Hospital (*n* = 65), Saint Gabriel Hospital (*n* = 131), Libikisi Reference Hospital (*n* = 107), and Maternity of Kintambo general referral hospital (*n* = 197). The participants at each health facility were selected using a simple random sampling technique until the required sample size at each health facility was obtained.

### Data collection

A structured questionnaire was used to collect data from pregnant women during ANC sessions. The questionnaire was initially developed in French and subsequently translated into the local language “Lingala” for non-native French speakers. Four interviewers, selected in advance and trained by the principal investigator in the methodology and use of the questionnaire, conducted the face-to-face interviews with pregnant women. The questionnaire was developed based on the findings of several other studies on pregnant women’s utilization of oral health care services [1, 7–9]. It contained information regarding the following variables of interest:

- Socio-demographic and economic characteristics:

- Socio-demographic status: age, education level, marital status, and parity.
- Socio-economic status: wealth index derived from a range of household assets (radio, television, landline telephone, personal computer, freezer, range cooker, furnaces, bed, sewing machine, clock, hoes, and house rental), housing conditions (availability of electricity and lamps), ownership of a means of transport (bicycle, motorcycle, and car), farmland, and domestic animals. The study participants were categorized based on their wealth index score, which was divided into quintiles of economic well-being [27].

- Knowledge of the relationship between oral health and pregnancy: the need of an oral consultation during pregnancy, awareness of the feasibility of dental care during pregnancy, importance of prevention and treatment of oral diseases by the dentist, relationship between oral health and the outcome of pregnancy, and relationship between hormonal changes during pregnancy and oral health.
- Oral health practices: tobacco use, alcohol use, frequency of tooth brushing, use of fluoride toothpaste when brushing, use of toothbrush for oral hygiene, duration of toothbrush use, and use of dental floss or interdental brush.
- Oral health status: gum bleeding, tooth decay, onset of perceived tooth decay, experience of dental pain or inflammation during the current pregnancy, difficulty chewing food, and oral health status since the onset of the pregnancy.
- Utilization of oral health care services:

- Status of oral health care utilization
- Reasons for utilization of oral health care services: reason for using oral health care services during pregnancy, oral problems motivating the utilization of oral health care services, pregnancy term during the oral health consultation, and management carried out during the oral health consultation.
- Reasons for non-utilization of oral health care services: cost of dental care, difficulty of movement, distance to the dental clinic, fear of complications with the pregnancy, lack of time, and no need for oral care during pregnancy.

To ensure the consistency and validity of the questionnaire, a pre-test was carried out on 5% of the final sample size in an environment similar to that of the present study. This was done by selecting pregnant women attending ANC services in a health facility on the outskirts of Kinshasa. Any necessary corrections were then made before the actual data collection by means of a structured interview.

Before collecting the data, we obtained authorization from the management of the selected health facilities and the administrative authorities of the localities hosting the survey sites. Pregnant women were then interviewed after the ANC visit, to ensure confidentiality. Interviews were only conducted with participants who had given verbal informed consent to participate in the study.

### Operational and term definitions

- Utilization of oral health care services: having consulted with an oral health care professional at least once since the start of the current pregnancy.
- Oral health knowledge: the ability to access, comprehend, and utilize fundamental oral health information and services to make well-informed decisions about their own oral health.
- Oral health awareness: concern and informed interest in oral health care.

### Data processing and analysis

The data collected were transcribed using an Epi-Data version 3.1 data entry mask. The generated database was exported to Microsoft Excel version 10 prior to cleaning, preliminary data processing, and analysis using R version 4.2.0.

Univariate analyses were performed to obtain descriptive statistics for the variables of interest, such as frequency and percentage (%) for the qualitative variables, and mean and standard deviations for the quantitative variable (age). Binary logistic regression analysis was employed to ascertain the factors associated with the utilization of oral health care services among pregnant women. The latter was designated as the dependent variable, with a value of 0 indicating no use and a value of 1 indicating use. All independent variables with *p* value < 0.20 in the bivariate logistic regression were considered as candidate factors for the subsequent forward stepwise multivariable model. The results were presented in the form of crude odds ratios (COR) and adjusted odds ratios (AOR), accompanied by their respective 95% confidence intervals (95% CI). Associations were considered statistically significant when the *p* value was less than 0.05. Multi-collinearity between the independent variables was assessed using the Variance Inflation Factor (VIF). Furthermore, the final model was evaluated for its fitness using the Hosmer-Lemeshow goodness-of-fit test.

## Results

A total of 500 pregnant women were included in this study. Their mean age was 28.4 (±5.3) years. The most representative age group was 25–34 years old (60.4%). The majority of the pregnant women had completed formal secondary education or above (86%), and were married (80.8%). More than half of these women had between 1 and 3 children (51.4%). The socio-economic level was approximately equal in our study (Table 1).

**Table 1:** Socio-demographic and economic characteristics of pregnant women.

Characteristics	*N* = 500
Age (years), mean (SD)	28.4 (5.3)
Age groups, *n* (%)	
<25 years	127 (25.4)
25–34 years	302 (60.4)
>35 years	71 (14.2)
Education level, *n* (%)	
Less than National high school leaving certificate	70 (14.0)
National high school leaving certificate or more	430 (86.0)
Marital status, *n* (%)	
Married	404 (80.8)
Single	96 (19.2)
Parity, *n* (%)	
Nulliparous	194 (38.8)
1–3	257 (51.4)
4 and more	49 (9.8)
Socio-economic level, *n* (%)	
Poorer	100 (20.0)
Poor	102 (20.4)
Average	99 (19.8)
Wealth	99 (19.8)
Wealthier	100 (20.0)

A minority of the pregnant women were aware of the need for oral health care during pregnancy (15%) and recognized the feasibility of dental care during this period (9.8%). Over half of the participants indicated that dental visits for routine and/or treatment purposes were not a priority during pregnancy (58%). Nine out of ten pregnant women were unaware of the link between oral health and the pregnancy outcomes (91.8%). A quarter of pregnant women were aware of the link between hormonal changes during pregnancy and oral health (25%) (Table 2).

**Table 2:** Knowledge of the relationship between oral health and pregnancy.

Variables	*n*	%
Did you know that you need an oral consultation during pregnancy?		
Yes	77	15.4
No	423	84.6
Are you aware of the feasibility of dental care during pregnancy?		
Yes	49	9.8
No	451	90.2
Is the prevention and treatment of oral diseases by the dentist important during pregnancy?		
Yes	175	35.0
No	290	58.0
Do not know	35	7.0
Did you know that there is a relationship between oral health and the outcome of pregnancy?		
Yes	29	5.8
No	459	91.8
Maybe	12	2.4
Did you know that there is a relationship between hormonal changes during pregnancy and oral health?		
Yes	126	25.2
No	369	73.8
Maybe	5	1.0

Table 3 shows that a minority of pregnant women (4.4%) were smokers, and over half of them (59.1%) reported that they had not ceased smoking since the commencement of their current pregnancy. Over a quarter of the pregnant women surveyed reported consuming alcohol (26.2%), with over half of them continuing to drink during pregnancy (58%). The vast majority of pregnant women (99.8%) reported using a toothbrush for oral hygiene, and 55.5% admitted to brush their teeth twice a day. More than eight out of ten pregnant women used a toothbrush up to three months before changing it (87.4%), used fluoride toothpaste when brushing their teeth (83%), and did not use dental floss or a brush for interdental hygiene (85%).

**Table 3:** Oral health practices among pregnant women.

Variables	*n*	%
Do you use tobacco (*n* = 500)?		
Yes	22	4.4
No	478	95.6
Did you stop smoking because of the pregnancy (*n* = 22)?		
Yes	9	40.9
No	13	59.1
Do you take alcohol (*n* = 500)?		
Yes	131	26.2
No	369	73.8
Did you stop drinking alcohol because of the pregnancy (*n* = 131)?		
Yes	55	42.0
No	76	58.0
Did you brush your teeth this morning (*n* = 500)?		
Yes	492	98.4
No	8	1.6
Frequency of tooth brushing (*n* = 492)		
Once a day	212	43.1
Twice a day	273	55.5
> Twice a day	7	1.4
Use of fluoride toothpaste during brushing (*n* = 500)		
Yes	415	83.0
No	45	9.0
Do not know	40	8.0
Toothbrush use for oral hygiene (*n* = 500)		
Yes	499	99.8
No	1	0.2
Duration of the toothbrush use (*n* = 499)		
≤3 months	436	87.4
>3 months	63	12.6
Use of dental floss or interdental brush (*n* = 500)		
Yes	75	15.0
No	425	85.0

Table 4 illustrates that nearly half of the surveyed pregnant women (47%) reported experiencing gingival bleeding since the onset of pregnancy, while 34% had experienced dental caries. Of these, only 7.6% were experiencing caries during the current pregnancy. A quarter of pregnant women reported experiencing dental pain or inflammation since the commencement of their pregnancy, and 22% have experienced difficulty chewing food. 45.4% perceived their oral health status as good since the onset of their current pregnancy.

**Table 4:** Oral health status among pregnant women.

Variables	*n*	%
Gum bleeding (*n* = 500)		
Yes	235	47.0
No	265	53.0
Tooth decay (*n* = 500)		
Yes	170	34.0
No	330	66.0
Onset of perceived tooth decay (*n* = 170)		
Before the current pregnancy	157	92.4
During the current pregnancy	13	7.6
Experience of dental pain or inflammation during the current pregnancy (*n* = 500)		
Yes	125	25.0
No	375	75.0
Difficulty chewing food (*n* = 500)		
Yes	110	22.0
No	390	78.0
Oral health status since the onset of the pregnancy (*n* = 500)		
Good	227	45.4
Average	196	39.2
Poor	77	15.4

Overall, 5% of pregnant women included in this study had utilized oral health care services since the commencement of their pregnancy. The primary reason for utilizing oral health care services was to address oral health problems (88%), particularly dental pain (64%), compared with routine visits (12%). Pregnant women were more likely to utilize oral health care services during the second trimester of pregnancy. The most common form of treatment was a medical prescription (52%) (Table 5).

**Table 5:** Utilization of oral health care services among pregnant women.

Variables	*n*	%
Utilization of oral services during current pregnancy (*n* = 500)		
Yes	25	5.0
No	475	95.0
Reason for using oral health care services during pregnancy (*n* = 25)		
Check-Up (routine visit)	3	12.0
Oral health problems	22	88.0
Oral problems that motivated the utilization of oral health care services (*n* = 25)		
Dental pain	16	64.0
Gum bleeding	6	24.0
Gum swelling	2	8.0
Halitosis	1	4.0
Pregnancy term during the oral health consultation (*n* = 25)		
1st trimester	10	40.0
2nd trimester	15	60.0
Management carried out during the oral health consultation (*n* = 25)		
Medical prescription	13	52.0
Dental fillings	5	20.0
Dental extractions	6	24.0
Taking X-rays	2	8.0

**Fig. 1:**
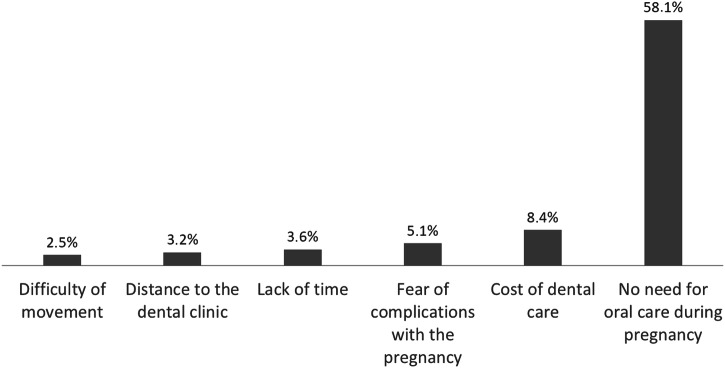
Reasons for non-utilization of oral health care services among pregnant women.

Figure 1 shows that the primary reason cited for the non-utilization of oral health care services by pregnant women was the lack of perceived need for oral care during pregnancy (58.1%), followed by the cost of dental care (8.4%), and the fear of complications with the pregnancy (5.3%).

Both univariate and multivariable binary logistic regression analyses were conducted to identify factors associated with the use of oral health care services by pregnant women (Table 6). The results of the multivariable logistic regression analysis indicated that the likelihood of utilizing oral health care services was 3.62 times higher among pregnant women who were aware of the need for oral care during pregnancy (AOR = 3.62, 95% CI: 1.42–9.26). Pregnant women who were aware of the importance of preventive care (routine visits) and treatment of oral diseases by a dentist during pregnancy were 4.94 times more likely to utilize oral health care services (AOR = 4.94, 95% CI: 1.70–16.73). Having experience oral health problems during the current pregnancy was significantly associated with the utilization of oral health care services by pregnant women (AOR = 3.13, 95% CI: 1.22–8.21).

**Table 6:** Bivariate and multivariable logistic regression on utilization of oral health care services among pregnant women.

Variables	Utilization of oral health care services		COR (95% IC)	AOR (95% IC)
	No, *n* (%)	Yes, *n* (%)		
Age groups				
<25 years	113 (95.8)	5 (4.2)	1.0	1.0
25–34 years	268 (95.7)	12 (4.3)	1.01 (0.37–3.23)	0.46 (0.13–1.76)
>35 years	59 (88.1)	8 (11.9)	3.10 (0.99–10.62)	2.23 (0.46–11.41)
Instruction				NI
Less than National high school leaving certificate	63 (96.9)	2 (3.1)	1.0	
National high school leaving certificate or more	377 (94.2)	23 (5.8)	1.92 (0.55–12.14)	
Marital status				
Married	351 (93.6)	24 (6.4)	6.00 (1.24–107.94)	5.64 (1.01–106.52)
Single	89 (98.9)	1 (1.1)	1.0	1.0
Socio-economic level				NI
Poorer	89 (95.7)	4 (4.3)	1.0	
Poor	91 (96.8)	3 (3.2)	0.73 (0.14–3.38)	
Average	86 (95.6)	4 (4.4)	1.01 (0.23–4.39)	
Wealthy	87 (93.5)	6 (6.5)	1.55 (0.43–6.22)	
Wealthier	87 (91.6)	8 (8.4)	2.09 (0.63–8.04)	
Parity				
Nulliparous	174 (97.2)	5 (2.8)	1.0	
1–3	224 (93.3)	16 (6.7)	2.51 (0.96–7.78)	1.65 (0.54–5.88)
4 and more	42 (91.3)	4 (8.7)	3.36 (0.80–13.19)	0.79 (0.12–4.83)
Tobacco intake				NI
Yes	19 (95.0)	1 (5.0)	1.0	
No	421 (94.6)	24 (5.4)	1.11 (0.22–20.31)	
Knowledge about the need for oral care during pregnancy				
No	382 (97.0)	12 (3.0)	1.0	1.0
Yes	58 (81.7)	13 (18.3)	6.96 (3.03–16.13)	3.62 (1.42–9.26) **
Knowledge about the importance of prevention and treatment of oral diseases by the dentist during pregnancy				
No	285 (98.3)	5 (1.7)	1.0	1.0
Yes	155 (88.6)	20 (11.4)	7.35 (2.91–22.44)	4.94 (1.70–16.73) **
Experience of oral health problems				
No	340 (97.1)	10 (2.9)	1.0	1.0
Yes	100 (87.0)	15 (13.0)	5.10 (2.25–12.06)	3.13 (1.22–8.21) *

*: *p* < 0.05, **: *p* < 0.01, 95% CI: 95% confidence interval, AOR: adjusted odd ratio, COR: crude odd ratio, NI: Not included in the multivariable logistic regression model (*p value* ≥ 0.20 in the bivariate logistic regression).

## Discussion

The objective of the present study was twofold: firstly, to ascertain the proportion of pregnant women in Kinshasa who utilize oral health care services; and secondly, to identify the factors associated with this utilization. The findings indicated that only 5% of pregnant women utilize oral health care services. This proportion is similar to those reported in other studies conducted in Africa, which demonstrates that pregnant women’s utilization of oral health care services remains a significant challenge in low-resource settings [13, 28–30]. The low rate observed in our study may be attributed to a number of factors, including a lack of awareness regarding the link between poor oral health and adverse pregnancy outcomes, as well as an inadequate comprehension of the importance of oral health care during this period [1, 10, 12, 31]. These findings are also supported by our other results, which indicate that a significant proportion of pregnant women lacked awareness of the relationship between oral health and the purpose of pregnancy, as well as the association between hormonal changes during pregnancy and oral health status. Furthermore, a relatively small proportion of pregnant women identified dental visits as a priority during pregnancy. There is a need to develop and implement initiatives that encourage routine oral and dental consultation with oral health counseling and check-ups as an integral and obligatory part of routine ANC.

Another likely reason for the low utilization of oral health care services by pregnant women in our study is the high cost of dental care. It is widely acknowledged that financial constraints play a pivotal role in determining whether or not individuals visit the dentist [28, 32]. In accordance with the WHO’s African Regional Strategy for Oral Health (2016–2025) [22], the universal health coverage initiative, launched by the Congolese government in several provinces since 2023, with a focus on free maternity and newborn care, provides an appropriate framework for public policy incentives to promote access and routine oral health care visits among pregnant women during ANC services. However, cost is not the sole determining factor in the utilization of oral health care services. The issue is also linked to the availability of dental materials and equipment, as well as the skills of the practitioners involved [33]. Consequently, the unequal distribution of oral health professionals and the insufficient and ineffective dental infrastructure within the primary health care system are the primary causes of this lack of access to or limited access to appropriate oral health care services for a large portion of the population. As a result, a significant number of oral diseases go undiagnosed and untreated [34]. In our context, the unequal distribution of oral health professionals among the different health facilities sampled in the four districts in Kinshasa did not have a significant impact on the level of oral health care utilization among antenatal care users (See Appendices 1 and 2). This situation indicates a potential knowledge gap among health professionals regarding oral health, which may limit their ability to promote optimal oral health care among pregnant women. Further studies on the knowledge, attitudes, and practices of health care workers concerning oral health among pregnant women would be beneficial.

Interestingly, we found that pregnant women who were aware of the need for oral health care and the importance of routine or treatment visits for oral diseases during pregnancy were more likely to use oral health care services than those who were not aware. These findings are consistent with existing literature that has demonstrated an association between oral health literacy before and during pregnancy and the frequency of oral health consultations among pregnant women [12, 35, 36]. It is evident that oral health education, regular follow-up by a dentist, and pregnant women’s self-perception of their oral health problems play a significant role in influencing the confidence of routine visits and treatment of oral diseases during pregnancy [35]. In our context, education and health promotion activities should include health workers informing patients attending ANC about the importance of oral health during pregnancy and requiring their patients to confirm oral and dental examinations during pregnancy.

Pregnant women who had experienced oral health problems during pregnancy were more likely to use oral health care services than those who did not. In fact, 88% of pregnant women surveyed sought care for oral health problems, particularly dental pain (64%), while the remaining 12% sought oral health care services through routine visits. There is a growing body of evidence that dental pain is the most common reason for dental consultation among the general population [37, 38], and among pregnant women in particular [39]. Another reason for dental consultation among pregnant women in our study was gingival bleeding. This finding is consistent with those of Mwangosi et al. in Tanzania and Kikelomo et al. in Nigeria [40, 41]. As evidenced by the literature [10, 13, 14], our results underscore the critical need for effective oral health educational programmes that are readily accessible to pregnant women. These would aim to improve oral health behaviors and increase in the prevalence of routine utilization of oral health care services during pregnancy. However, it should be noted that whilst educational programmes aim to improve behaviors, it is also important to ensure that oral health care services are made as accessible as possible.

### Limitations and strengths of the study

There are some limitations that can be considered in this study. Due to its cross-sectional nature, it was not possible to draw any conclusions regarding causality. Secondly, as is the case with any study conducted in a health setting, and as a consequence of the use of convenience sampling, the results may not be generalized to all pregnant women in the study area. Thirdly, there is a possibility that respondents may have been subject to social desirability and recall bias in their self-reporting of information related to the utilization of oral health care services. Nevertheless, to the best of our knowledge, this is the first study to analyze the current status and the key factors underlying the utilization of oral health care services in pregnant women in the DRC, which accounts for one of the highest maternal and neonatal mortality rates in Africa [16–18]. As documented in the extant literature, poor oral health during pregnancy can be detrimental to the maternal health and also to the well-being of the infant [1, 2, 42, 43].

## Conclusion

The results of this study indicate that the utilization of oral health care services by pregnant women in Kinshasa is alarmingly low. A significant proportion of pregnant women demonstrated a lack of awareness regarding the link between oral health and pregnancy outcomes, as well as an underestimation of the importance of oral health care during pregnancy. Given the importance of oral health care during pregnancy, it is imperative to implement tailored public health initiatives to facilitate collaboration between ANC providers, dentists, and other health professionals. This will ensure the establishment of a sustainable referral system that includes oral and dental consultations, oral health counseling, and check-ups as an indispensable component of routine ANC.

## Supplementary information

Supplementary material

Dataset 1

## Data Availability

All data generated or analyzed for this study are included in this published article.
