# Supplementary material for: Frequency and factors associated with the utilization (curative and preventive) of oral health care services among pregnant women in Kinshasa, Democratic Republic of Congo
Source: BDJ Open. 2025 Feb 14;11:15. doi: 10.1038/s41405-025-00308-w (PMC11828865; doi:10.1038/s41405-025-00308-w)

Appendix 1. Distribution of dentists according to health facilities

**Health facilities**	**n**	**%**
Libikisi Reference Hospital	5	9.8
Saint Gabriel Hospital	1	2.0
Sino-Congolese Friendship Hospital	15	29.4
Maternity of Kintambo general referral hospital	30	58.8

Appendix 2. Frequency of oral health care utilization among pregnant women according to health facilities

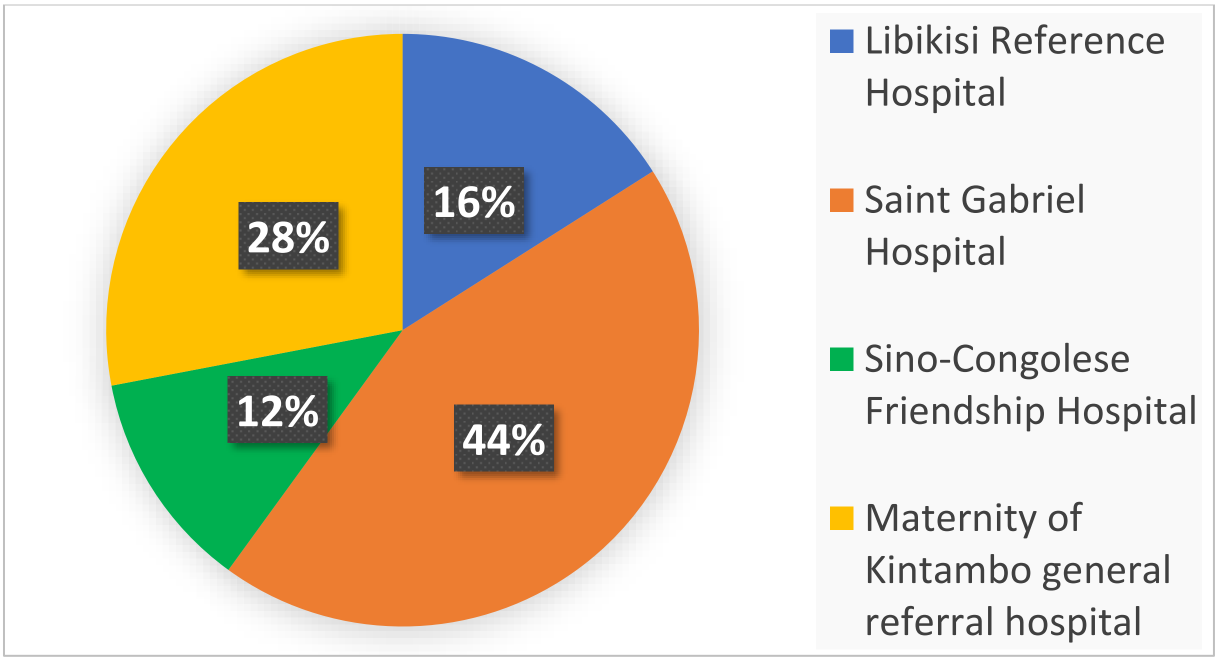

Supplement: Supplementary file 1 — Supplementary material [file 41405_2025_308_MOESM1_ESM.pdf]
